# Evaluation of Genetic Diversity Based on Microsatellites and Phytochemical Markers of Core Collection of *Cymbopogon winterianus* Jowitt Germplasm

**DOI:** 10.3390/plants11040528

**Published:** 2022-02-16

**Authors:** Sunita Munda, Raktim Jyoti Saikia, Twahira Begum, Sangeeta Bhandari, Ankita Gogoi, Neelav Sarma, Raghu Tamang, Mohan Lal

**Affiliations:** 1 CSIR-North East Institute of Science and Technology, Jorhat 785006, Assam, India; mundasuni26@gmail.com (S.M.); raktimjyotis91@gmail.com (R.J.S.); twahira.begum24@gmail.com (T.B.); bhandarisangeeta46@gmail.com (S.B.); ankitagogoi2014@gmail.com (A.G.); neel94sarma.ns@gmail.com (N.S.); raghutamang22@gmail.com (R.T.); 2 AcSIR-Academy of Scientific and Innovative Research, Ghaziabad 201002, Uttar Pradesh, India

**Keywords:** *C. winterianus*, molecular diversity, phytochemical diversity, N-J Method, Euclidean distance

## Abstract

*Cymbopogon winterianus* Jowitt is an industrially important crop due to its value in the aromatic, perfumery and pharmaceutical industries. In this study, 72 accessions of *C. winterianus* were selected for molecular diversity analysis using SSR markers. It revealed a total of 65 polymorphic alleles showing an average of 68.10% polymorphism. The best SSR primer with competency in discriminating the germplasm was 3CM0506 with PIC (0.69), MI (0.69) and Rp (3.12). Genetic variation was studied between Assam, Manipur, Meghalaya and Arunachal Pradesh populations. A dendrogram based on the Neighbour-Joining Method showed clustering of germplasm on the collection site. A total of six relevant genetic populations were identified through a structure harvester software analysis. Moreover, a dendrogram based on similarity, complete linkage and Euclidean distance was also elucidated differentiating the genotypes with respect to the major phytochemical constituents of the essential oil. GC-FID and GC-MS analyses of the essential oil of the 72 germplasms revealed citronellal content from 2.58–51.45%, citronellol from 0.00–26.39% and geraniol from 0.00–41.15%. This is the first molecular diversity report with 72 accessions of *C. winterianus* collected from the NE region using 28 SSR primers as well as their diversity based on phytochemical markers. This diversity computation will help with acquisition of the knowledge and relationship among each individual accession leading to the development of improved and essential oil component-rich cultivars.

## 1. Introduction

Genetic diversity analysis is a major step for developing plant breeding programmes [1]. A better understanding of the genetic relationship amongst plant species’ germplasms, both cultivated and wild, is necessary to formulate strategies for the breeding, conservation and utilization of the genetic resources [2] of a crop’s wild relatives, especially those with a primary gene pool [3].

One genus containing profound genetic diversity is the *Cymbopogon* species, belonging to the Poaceae family, which contains approximately 140 species of heterogeneous plants due to its cross-pollinative nature [1,4,5]. These species are generally distributed in tropical and subtropical regions of the world with an availability of 45 species grown in India [1,6,7]. These species are characterised by an aromatic essential oil, bearing many bioactive compounds that have a high pharmaceutical importance and economic value [8]. 

One of the important species of this genus, *Cymbopogon winterianus* Jowitt, popularly known as Java citronella, is a perennial grass that propagates by vegetative means [7,9,10]. The crop is cultivated for the extraction of essential oil, which has aromatic and medicinal properties [8]. The leaf essential oil has vast use in the perfumery, cosmetics and flavouring industries [10,11]. Due to the presence of this fragrance, the leaf essential oil is used in the food industry [12]. The Java citronella essential oil is used as an antiseptic, antispasmodic and diuretic drug [13] and possesses analgesic and anticonvulsant activities [14]. Moreover, the anti-microbial activity of *C. winterianus* has been reported against different pathogenic bacteria and fungi [13,14,15]. It has also been used over the ages in Brazilian and Indian folk medicines [16].

The *Cymbopogon winterianus* leaf essential oil is marked by the presence of citronellal, citronellol and geraniol as the major compounds [10,15,17]. These constituents can alternatively be used as phytochemical markers for the identification of the species. Phytochemical variation in the essential oil of this species has been observed due to the genetic makeup of the genotype, as well as due to environmental factors [18], which was also reported in other wild species where different chemotypes were observed depending on multiple environmental factors (e.g., vegetation type, topographical parameters, climatic conditions and pedological features) [19]. Various previous studies have reported that the *C. winterianus* essential oil, rich in these compounds, was responsible for the bioactivity [20]. Citronellol is a monoterpene alcohol that has antimicrobial, anticonvulsant, anticancer, anti-inflammatory and analgesic properties [21]. Moreover, citronellol has been used in the fragrance industry, in perfumery, as an insect repellent and mite attractant [21,22]. Citronellal is an aldehyde, which gives *C. winterianus* its characteristic lemon-like fragrance. It has an effective mosquito repellent activity, and thus is used as the active constituent of different mosquito repellent products [23]. It has been used in the pharmaceutical industry due to its anti-microbial, antinociceptive, anti-inflammatory and anticancer activities [24]. Another constituent of the *C. winterianus* essential oil is geraniol, which is an aromatic compound with a rose-like fragrance [25]. Geraniol is effective at preventing certain types of cancer, and possesses antimicrobial, anti-inflammatory, antiseptic and insect repellent activities [26,27]. It is also used mainly in the fragrance industry as well as the cosmetic industry [28]. Both citronellal and geraniol are the starting material for the synthesis of many aromatic compounds [29,30]. 

Over the course of years, Java citronella has been introduced to different geographical locations and climatic conditions, which has led to further genetic differentiation amongst the germplasms through human selection, as well as domestication [31]. A wide variation in terms of essential oil, as well as morphological characters, has been observed in these species, but these are greatly affected by environmental fluctuations and the growth stage [4,32]. Moreover, genetic diversity in the crops is also reduced due to changing agricultural practices, deforestation, overgrazing, and urbanization. In addition to this, conservation and management of the crops is a slow and costly process because of several developmental stages in the crop and the environmental conditions affecting its growth. Molecular markers have helped with combating this problem by elucidating information related to the diversity, which is useful for planning different conservative measures. A rich source of genetic diversity is a prerequisite for introducing novel traits into the crop [9,33]. Therefore, an analysis of genetic divergence through molecular markers has an edge over the morphological characters because they are not affected by external factors [34]. For an assessment of the genetic variations within the germplasm, DNA-based molecular markers like RAPD, ISSR, and SSR markers are widely used. Many studies were reported on the genetic diversity of *Cymbopogon winterianus* using RAPD and ISSR molecular markers [4,31,35], but the number of germplasms used in these studies was very limited. Additionally, one of the drawbacks of RAPD marker is its low reproducibility [36]. Currently, SSR markers are considered useful because they are highly sensitive to genomic polymorphism [1], and they are robust and inexpensive [37]. Phylogenetic assessment and genetic diversity were successfully studied in many crops using microsatellites (SSR) [38,39,40]. Microsatellites are co-dominant markers; they are locus specific and have the capacity to detect a high level of polymorphism in the genome [41,42]. Kumar et al. (2009) studied the genetic diversity of the *Cymbopogon* species using SSR markers because of their ability to differentiate between the species precisely [32]. This will help in the development of future strategies for the improvement and commercial cultivation of this industrially important plant species. The genome mapping of complex traits and the selection of suitable genes for breeding can also be studied with this molecular tool [43,44]. Reports on the genetic divergence of different *Cymbopogon* taxa were performed [36], but very scanty information is available on the *C. winterianus* germplasm.

Hence, the present study aimed to estimate the level of divergence existing among and within the population of this species using SSR marker. The study of diversity based on phytochemical and molecular markers together is regarded as an appropriate measure to conserve and screen the elite genotypes for improvement of the crop and increase the gene pool [45,46,47,48]. This would also help in the identification and crossing of parents with desirable traits, subdivided into clusters, for the development of a superior germplasm with more yield-related traits. Segregating populations would also aid an increase in the recombinant combination in the gene pool. To the best of our knowledge, this is the first report on the phytochemical, as well as molecular, diversity of 72 accessions of *C. winterianus*.

## 2. Results and Discussions

All 72 accessions were submitted to ICAR-National Bureau of Plant Genetic Resources (NBPGR), New Delhi, to obtain the Indigenous collection number from IC-0626991 to IC-0627065 represented in Table 1. 

This helps in the conservation of genetic resources; utilization of the germplasm for screening of elite genotypes; for the development of superior genotypes by crossing or genetic modification of the genes responsible for economic yield; and for the transformation of genotypes with disease resistant, stress and pest tolerance traits. It is the most effective way of conserving all genes present in the germplasm for a long duration and can be regenerated whenever required. This can serve as a resource house for essential and newly identified genes, thereby creating a genetic information library. 

### 2.1. SSR Primer Competency

Initially, 45 SSR primer pairs were selected based on the literature review of the genus *Cymbopogon* and procured from Bioserve Biotechnologies (India) Private Limited, Hyderabad. Primer testing was performed using 45 SSR primer pairs, of which 28 primer pairs were found to be efficient for the analysis, showing good amplification and clear reproducible patterns. The screened primer sequences, polymorphism percentages, PIC, MI and Rp are indexed in Table 2. 

**Figure 1:**
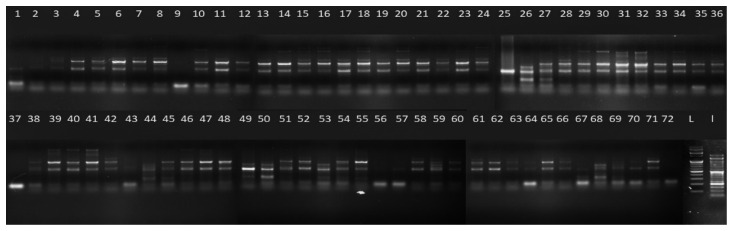
Gel image of bands formed for 72 accessions of *C. winterianus* using primer 3CM0506.

The amplified bands obtained showed 2–5 alleles/locus. In total, 92 alleles were produced from 28 SSR primers, of which 65 were polymorphic, and the remaining was monomorphic. The polymorphism percentage of the primers ranged from 50% to 100% with an average polymorphic percentage of 68.10% (Table 2). The primers 3CM0506, 14CM3132, 30CM056 and 34CM014 represented 100% polymorphism. Previously, a RAPD and ISSR analysis of *C. winterianus* accessions collected across West Bengal (India) showed polymorphism of 74.07% and 47.5%, respectively [1], while 75.11% polymorphism was reported in other species of *Cymbopogon* [49] and 81.33% in RAPD analysis [50]. Similarly, 90.68% and 69% polymorphism were reported in ISSR analysis, while 88.62% and 62.93% polymorphism were reported in RAPD analysis [35,51]. An EST-SSR analysis in *Cymbopogon* species showed ~81% polymorphism [32]. In the present diversity analysis of *C. winterianus* using SSR marker, an average of 68.10% polymorphism was observed. The level of polymorphism was low compared to the other studies since the present study was performed on the same *Cymbopogon* species collected from different geographical locations. Therefore, they are likely to be more related to each other. In addition to this, PIC, MI and Rp values were also evaluated to check the competency of the screened primers. The mean PIC (polymorphism information content) values for all the loci of a particular primer varied from 0.16 (17CM3738) to 0.69 (3CM0506). A high value of PIC is an indication of more variation in the alleles and also describes the efficiency of the primers [52]. Therefore, from the observed data, 13 of the loci (PIC ≥ 0.50) can be considered more informative than the others. The PIC value >0.50 depicts the high efficiency of the primers [53]. Based on this standard value, 13 of the 28 SSR primers can be considered highly efficient for discriminating the *C. winterianus* accessions. In a study performed on different *Cymbopogon* species, 19 out of 20 primers were found to be very informative [32] with PIC > 0.50. The primer efficiency was checked by further calculating the marker index (MI) value, where the primer 3CM0506 had the highest MI of 0.69, and the lowest were the primers 5CM1112 and 17CM3738 with a 0.11 marker index. The average MI was found to be 0.34 per primer. Similarly, the resolving power (Rp) of a primer is the ability to discriminate between genotypes. Rp values varied from 0.01 (5CM1112) to 3.12 (3CM0506) with an average of 0.96/primer. Based on the data evaluation, the most efficient primer among the screened primers was the 3CM0506, with PIC (0.69), MI (0.69) and Rp (3.12). The gel image of the primer 3CM0506 differentiating 72 *C. winterianus* accessions is shown in Figure 1. The primer 5CM1112 could be considered ineffective due to its inability to differentiate variation among the genotypes with low PIC (0.33), MI (0.11) and Rp (0.01).

### 2.2. Intra- and Inter-Population Genetic Variation

The genetic variability parameters, i.e., number of observed alleles (na); number of effective alleles (ne); Nei’s gene diversity (h); Shannon’s information index (I); genetic diversity in the population (Ht); genetic diversity within the population (Hs); genetic differentiation degree (Gst); and gene flow (Nm), were calculated at the population level (Table 3). 

The genotypes were divided into four populations: Pop 1 (Assam), Pop 2 (Manipur), Pop 3 (Meghalaya) and Pop 4 (Arunachal Pradesh). The parameters (na, ne, h and I) were highest for the Assam population (1.71, 1.42, 0.24 and 0.36), followed by the AP population (1.58, 1.34, 0.20 and 0.31), the Manipur population (1.40, 1.23, 0.13 and 0.20) and the Meghalaya (1.18, 1.13, 0.07 and 0.11) population. The Shannon’s diversity index revealed the genetic diversity within and among the population. The highest variation within a population was observed in Pop 1, followed by the variation in Pop 4, Pop 2 and then Pop 3. All populations showed moderate genetic diversity with 70.65% polymorphism (Pop 1), 57.61% (Pop 4), 40.22% (Pop 2), except for Pop 3 with only 18.48% polymorphism. The low polymorphism observed in the genotypes collected from Meghalaya could be due to the smaller number of germplasms used in the study.

The diversity of a species depends on the frequency of heterozygosity (Ht). The total species diversity among the population was found to be 0.22 ± 0.03, and within the population (Hs) was 0.16 ± 0.02. Further, the gene differentiation coefficient (Gst) was 0.25 with a gene flow of 1.48, indicating significant diversity in the population. All of these results confirmed the genetic variation in the populations because the Nm was higher than the threshold value (Nm ≤ 1.0) [54]. Similar results were also reported in the *Cymbopogon* species, which showed a gene flow of 2.58 (ISSR) and 2.20 (RAPD) [35]. Moderate heterozygosity (Ht) and gene differentiation coefficient (Gst) suggested moderate genetic variation among the population. Our results were in accordance with earlier reports [42,55]. 

### 2.3. Cluster Analysis

**Figure 2:**
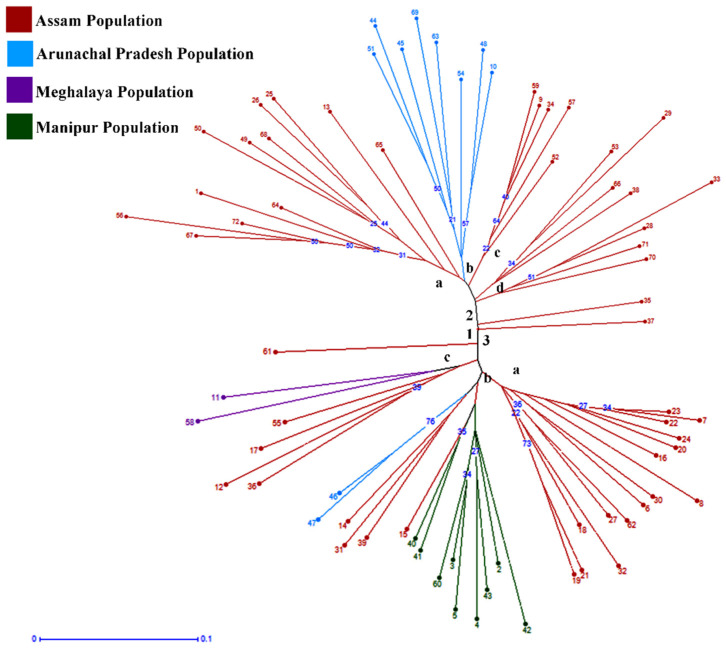
Dendrogram based on N-J Method representing three clusters in *C. winterianus* germplasm. Numbers indicated in the dendrogram represent serial number of *C. winterianus* accessions.

Clustering of the genotypes was constructed based on the Neighbour-Joining Method (N-J) using a pairwise distance matrix, which generated three major clusters (Figure 2). Cluster I comprises of only one accession, while Cluster 2 and Cluster 3 comprise of 34 and 37 accessions, respectively. Cluster 2 is further subdivided into Cluster 2a, consisting of 12 accessions collected from Assam; Cluster 2b, consisting of accessions collected from Arunachal Pradesh; and Clusters 2c and 2d, both consisting of accessions collected from the Assam region. Similarly, Cluster 3 is also divided into three sub-clusters with one outgroup, i.e., Accession No. 16 (IC-0627058), collected from North Lakhimpur, Assam. Cluster 3a consists of accessions collected from Assam, and Cluster 3b consists of all the accessions collected from Manipur as well as four accessions from Assam and two accessions of Arunachal Pradesh. The dendrogram demonstrates that the clustering of accessions was dependent on their geographical collection site with the exception of two accessions from Arunachal Pradesh that were clustered separately in a different group. The reason for this may be either due to the migration of the same genotype from one location to another or due to the inability of the primers to discriminate them. The grouping of genotypes based on the N-J Method is preferred because it is rapid, reliable and produces an unrooted phylogenetic tree based on the minimum evolution criterion [56]. 

**Figure 3:**
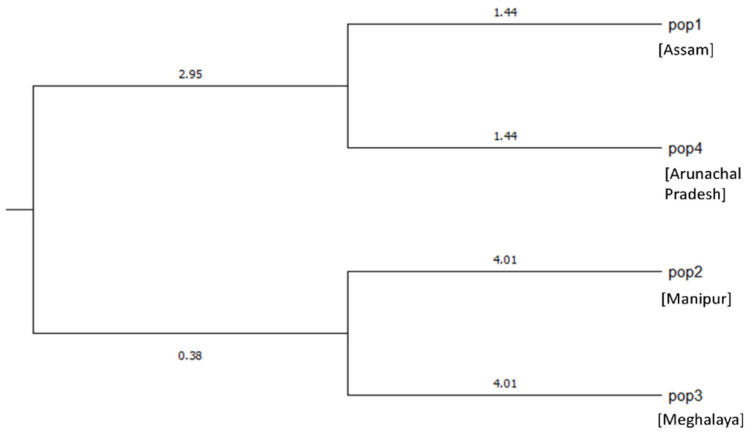
Dendrogram based on Nei’s genetic distance between populations of *C. winterianus*.

Jaccard’s pairwise coefficient of similarity depicted a minimum genetic similarity (0.485) between IC-0627064 (Cluster 3c) and IC-0627012 (Cluster 2a), as well as between IC-0627049 (Cluster 3a) and IC-0627048 (Cluster 3a), representing the minimum variation (0.928) among the genotypes. This pairwise similarity matrix depicted less variation among the genotypes. This may be due to the non-cross-pollinative nature of *C. winterianus*, due to rare flowering unlike the other *Cymbopogon* species [4]. Further, Nei’s genetic identity and the genetic distance were calculated between the four populations. The dendrogram constructed indicated the highest genetic identity between Pop 1 and Pop 4 and a maximum distance between Pop 4 and Pop 3 (Figure 3). 

Nei’s genetic identity and the genetic distance between the four populations showed low variation, indicating the gene exchange or duplication of germplasms between adjacent geographical populations [57]. In *Cymbopogon winterianus*, due to the presence of very rare flowering, the gene exchange is not carried out by pollens or seed. Therefore, we can interpret that the factors affecting the gene flow are mainly due to human interference, such as genetic swamping, genetic rescue, hybridization and urbanization.

**Figure 4:**
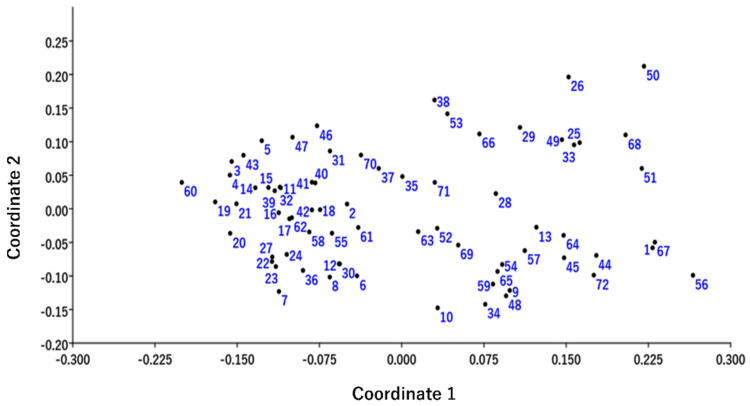
Principal component analysis of all accessions of *C. winterianus* collected from different regions of NE India.

The basis of a multivariate analysis is a principal component analysis which is an approximation of the data table provided by the analysis [58]. A field representation of variability can be provided by utilizing the PCA. The PCA is very useful for determining the similarity of samples as the non-similar samples become further apart in the presentation [36]. Population structure analysis and the PCA are used widely to visualize the structure of the data [59]. Therefore, the PCA was performed for the 72 accessions of *C. winterianus* to check the variability and relationships among them, which is represented in the biplot (Figure 4). 

The Eigen values are higher in the first three groups (1.91, 0.99 and 0.69), which show a greater contribution to the explanation of variances among the accessions. The total cumulative variance observed for the fourteen principal components accounted for 69.33% of the variance, of which 16.09%, 8.36% and 5.77% of the variance was contributed by the first, second and third principal components, respectively (Table 4). 

A cluster analysis along with the PCA based on molecular marker data help in the extraction of maximum information if the first three principal components account for more than 25% variance [60]. The present analysis revealed that the first three components contribute to 30.22% variance, which is in accordance with the previous study. The PCA plot resembled the cluster formed in the dendrogram, although some diversions of the accessions were observed on the PCA plot (Figure 4). 

*C. winterianus* is a plant of industrial importance globally; therefore, the improvement of this crop through breeding programme is very essential. Germplasm collection, along with their variability, is required for any genetic improvement of the crop. However, very scanty evidence on the intra- and inter-specific relationships and genetic diversity within the *C. winterianus* germplasm is available; therefore, the present study will add new information for researchers and breeders.

### 2.4. Population Structure

**Figure 5:**
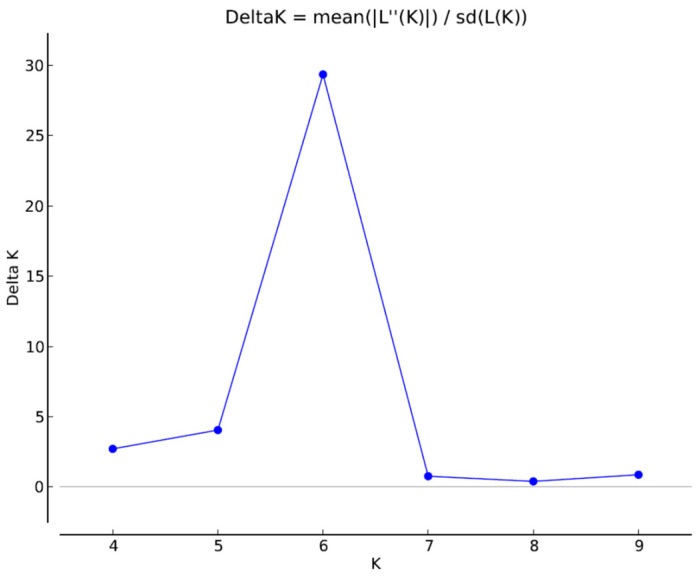
Estimation of relevant number of genetic populations in 72 accessions of *C. winterianus*.

**Figure 6:**
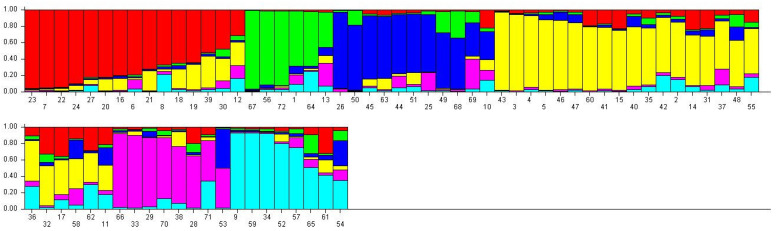
Model-based population structure analysis of 72 accessions of *C. winterianus*.

A total of six appropriate genetic populations was identified through a structure harvester software analysis (Figure 5 and Figure 6). The accessions that showed a probability score of more than 0.80 can be deemed genetically pure accessions, while a score of less than 0.80 can be considered as homogenous accession. The mean Fst values for Populations I, II, III, IV, V, and VI are 0.6023, 0.5092, 0.3296, 0.5244, 0.3483 and 0.4550, respectively. The allele frequency divergence among a population was computed using point estimates of P, which are presented in Table A1. The population structure study indicated the genetic differentiation of the *C. winterianus* accessions, which suggested that the SSR primers used in the study were suitable for population structure studies.

### 2.5. Analysis of Molecular Variance (AMOVA) of C. winterianus Accessions

AMOVA was used to interpret the difference in population using molecular markers [61]. Analysis of the *C. winterianus* accessions showed a high variance within the population (75%) and significantly less variance among the populations (25%), indicating a continuous gene exchange among the populations (Table 5). 

AMOVA results showed an 80% variation within the populations in RAPD, and a 79% variance in ISSR analysis as well as significantly less variation were found among the population of the *Cymbopogon* species with 20% (RAPD) and 21% (ISSR) [35]. It was reported that when an intra-specific variation is compared to an inter-specific variation, the presence of the population structure can be predicted [62]. Many factors, such as the geographical location, genetic drift, gene flow, mating system, long-term evolutionary history and wind-pollinated, long-lived outcrossing in species were responsible for genetic diversity [63]. In addition to this, population size plays an important role for the precision of work and avoiding skewed results and errors [64]. Therefore, the highest intra-specific diversity was observed in Pop 1 (Assam) and lowest in Pop 3 (Meghalaya). According to the genetic theory of population, an increase in diversity helps species’ potential for adapting to a changing environment [65]. Therefore, it is necessary to broaden the knowledge of genetic bases because a loss of heterogeneity might affect the feasibility of a population leading to species extermination [66].

### 2.6. Genetic Diversity Based on Phytochemical Analysis of the Biomarkers

**Figure 7:**
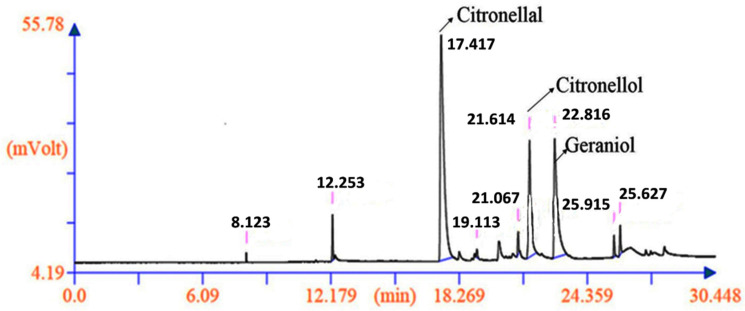
Chromatogram representing GC analysis of the best line (IC-0627062) with high phytochemical contents (citronellal, citronellol and geraniol).

The qualitative and quantitative analyses were performed using GC-FID and GC-MS, which represented citronellal, citronellol and geraniol as the major compounds. These are the main biomarkers of the *C. winterianus* essential oil; therefore, only these three compounds were considered for a chemical diversity analysis. The maximum citronellal (51.450%), citronellol (26.389%) and geraniol (41.146%) were present in the genotype IC-0626993, IC-0627002 and IC-0627004 respectively This would serve as a good source for processing high grade pharmaceuticals, cosmetics and value-added products [30,67]. In all 72 accessions, the citronellal content differed from 2.578% to 51.450%, the citronellol content from 0.000% to26.389% and the geraniol content from 0.000% to 41.146% (Table 1), proving diversified germplasm. Previously, a new variant of *C. winterianus* with 1.2% essential oil and 35% citronellal content was developed through mutation breeding and registered as INGR-16021 [10,11]. The chemical profiling of *C. winterianus* showed citronellal content of more than 43% in the M 6–10 cultivar developed through mutagenesis, citronellol content of 15.3% in Mandakini, and geraniol content of 60% in Medini, identified through phenotypic selection [31]. Similarly, the present study demonstrated IC-0626993 as the best line for the citronellal content with 51.45%, while the genotypes IC-0627002 and IC-0627004 were rich in citronellol (26.38%) and geraniol (41.15%), respectively. All variants possessing high levels of the three measured constituents were identified through selection that may be further developed for superior lines. From the quantitative and qualitative analyses of the essential oil, IC-0627062 was identified as the ideal genotype with a commendable amount of the phytochemicals (citronellal: 48.48%, citronellol: 9.25% and geraniol: 20.48%). A GC chromatogram of germplasm (IC-0627062) is shown in Figure 7. 

**Figure 8:**
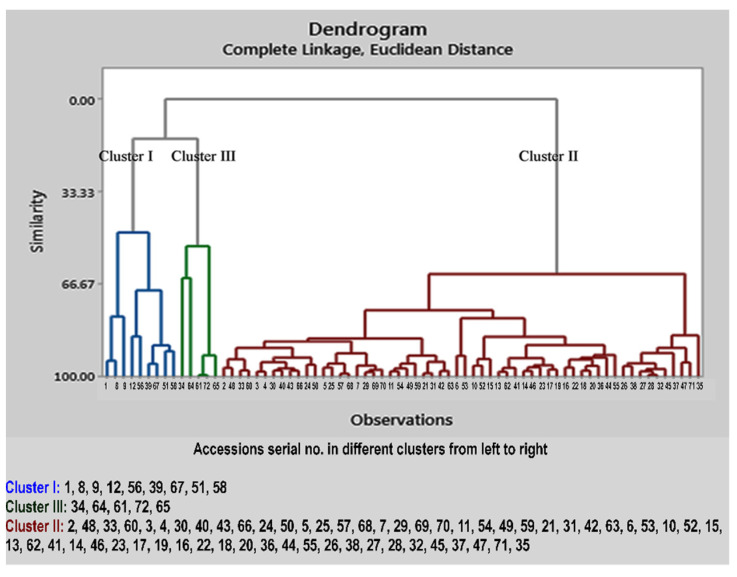
Dendrogram constructed based on the phytochemical diversity of *C. winterianus* accessions, representing three major clusters. The numbers indicated in the dendrogram represent the serial number of the *C. winterianus* accessions.

Apart from the molecular diversity analysis, a variation based on the essential oil constituents was performed by different researchers on different species of the genus *Cymbopogon* [36]. However, in the current study, diversity based on the phytochemical analysis of the essential oil was investigated in the same species of *C. winterianus*. Therefore, using these biomarkers, a dendrogram was constructed based on similarity, complete linkage and the Euclidean distance, which formed three clusters. Cluster I comprises nine accessions; Cluster II comprises fifty-eight, and the remaining five accessions come under Cluster III (Figure 8). 

**Figure 9:**
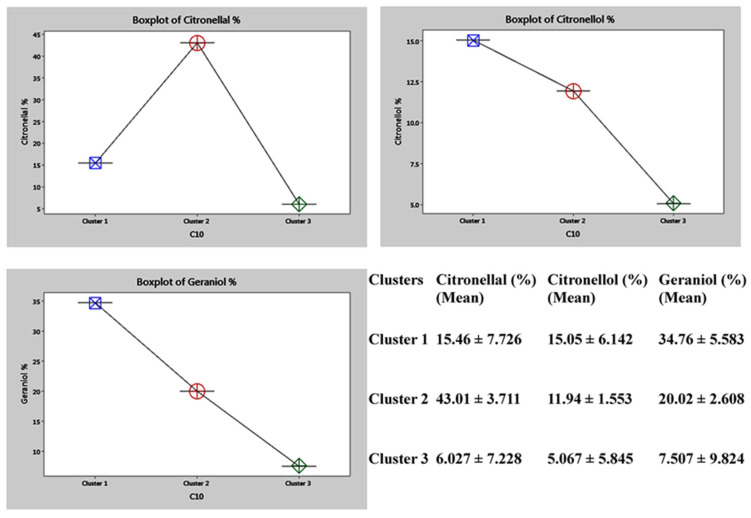
Boxplot of the major phytochemical markers along with their cluster mean values.

A boxplot graphical representation of these markers depicts that Cluster I involves the accessions with high citronellol and geraniol contents, while the Cluster II accessions were high in citronellal content. Cluster III comprises the accessions with low levels of the compounds measured (Figure 9). 

Previously, it was reported that *C. winterianus* is different from *C. nardus* in the context of the major constituent of the essential oil because the former contains citronellol as the major component, while the later contains geraniol [68]. However, it was also proved that the production of phytochemicals might be due to genetic makeup and geographical variations [18,47]. The analysis of both phytochemical and molecular markers revealed different clustering, probably due to the incorporation of a locus-specific SSR marker concerning the genes not responsible for phytochemical biosynthesis [32]. 

## 3. Materials and Methods

### 3.1. Plant Materials

A total of 72 accessions of Java citronella were collected from different states (Assam, Arunachal Pradesh, Manipur and Meghalaya) in Northeast India. The GPS locations of the collected accessions were previously reported [69]. The plants were identified by the plant breeder of the Medicinal, Economic and Aromatic Plant (MAEP) Group of the CSIR-North East Institute of Science and Technology (NEIST). The accessions were planted during *kharif* 2016 and maintained at the NEIST Institutional experimental farm in Jorhat. The latitude and longitude of the experimental farm were recorded using the WGS84 geographical system as 26°44′15.6948′′ N and 94°9′25.4628′′ E, respectively. The pH of the experimental soil was 4.9 with a sandy loam texture. The NPK (nitrogen, phosphorus and potassium) available were 224, 115 and 142 kg/ha, respectively. 

### 3.2. Genetic Diversity Analysis Based on Molecular Marker

#### 3.2.1. Genomic DNA Extraction

The tender leaves of the *C. winterianus* germplasm were collected separately in different zip lock bags from the experimental farm of CSIR-NEIST, Jorhat. The leaf samples were cleaned, lyophilised for 48 h at −40 °C and tissue lysed in Tissue Lyser (Qiagen, Germany) using liquid nitrogen gas (N_2_) for 150 s at a frequency of 25 Hz. Genomic DNA extraction was carried using HiPurA™ Plant DNA Isolation Kit by CTAB method (Cetyl trimethylammonium bromide). The purity of the extracted DNA was checked using 0.8% agarose gel, and the bands observed were detected in a gel-documentation system (Eppendorf, Germany). The concentration was quantified using 3 µL of the stock DNA in a Nano Bio Spectrophotometer (Eppendorf, Germany) at λ_260_/ λ_280_ ratio. The stock DNA was further diluted to 30 ng/µL for PCR analysis using SSR marker.

#### 3.2.2. SSR Primers–PCR Analysis

A total of 42 pairs of microsatellite (SSR) primers were screened, out of which the best amplifications were observed in 28 primer pairs, for the analysis of genetic diversity in the *C. winterianus* germplasm (Table 2). The reaction mixture for an SSR–PCR analysis consists of 6μL of working DNA (30 ng/µL), 1.6 μL each of forward and reverse primer (Bio Serve Biotechnologies, Hyderabad, India), 8.5 μL of 1X Hi-Chrome PCR Master Mix (Hi-Media, India) and 2.3 μL of double distilled water, making the total volume 20 μL. A Prima-96 (Hi-Media, India) thermocycler was used for amplification of the product with PCR programming of 94 °C (initial denaturation) for 3 min, then 35 cycles of denaturation for 30 s at 94 °C, annealing for 60 s at primer melting temperature (Tm) ±5 °C, extension for 90 s at 72 °C and final extension for 10 min at 72 °C. The amplified product along with 100 and 50 bp ladder (Hi-Media, India) was observed using 2% agarose gel in a 1× TBE buffer. Additionally, aliquots (15 µL) were also separated using 8% Polyacrylamide Gel Electrophoresis (PAGE). The acrylamide gel was stained with 0.5 mg/mL SYBR solution which is considered a safe stain for 25–30 min. The DNA bands in the gel were then visualised in a gel documentation system. Electrophoresis was run for 1 h at 100 constant voltages in the 1X TBE buffer.

#### 3.2.3. Statistical Analysis of Data

The amplicons formed were observed in the gel documentation system and were scored to obtain the genetic variation. The Neighbour-Joining tree, distance matrix and principal coordinate analysis (PCA) were constructed based on Jaccard’s pairwise distance matrix using Darwin software version 6.0. Based on the polymorphism of the bands, the PIC (Polymorphic information content) percentage was calculated as follows: PIC = 1 − Σpi^2^, where Pi is the frequency of the ith allele [53]. Marker index (MI) was calculated using the formula MI = EMR × PIC where the effective multiplex ratio (EMR) is the product of a number and a fraction of the polymorphic loci [70,71], whereas resolving power (Rp) is the summation of the band informativeness [*I_b_* = 1 − [2 × |0.5 *− p*|] and *p* is the proportion of individuals containing the band [71]. The genetic diversity variables, such as genetic differentiation degree (Gst), genetic diversity in the population (Ht), genetic diversity within the population (Hs), Shannon’s information index (I), number of observed alleles (na), number of effective alleles (ne) and Nei’s gene diversity (h), were calculated using the POPGENE (Version-1.31) software package [72]. The inter-specific and intra-specific genetic diversities were determined using analysis of molecular variance (AMOVA) with the help of GenAlex software Version 6.5 [73]. The genetic relationships among the 72 accessions were established using 28 SSR primers and utilizing a model-based population structure, which was performed using STRUCTURE software Version 2.3.4. The software was run multiple times by setting k (the number of populations) from 3 to 10, and the length of the burn-in period and number of Markov Chain Monte Carlo (MCMC) replications were set at 100,000 for each run for all 72 accessions in order to evaluate the number of populations. An online tool called Structure Harvester was used to calculate the most probable genetic population groups from the study. 

### 3.3. Quantitative and Qualitative Analysis of the Citronellal, Citronellol and Geraniol (Biomarkers) by GC-FID and GC-MS

#### 3.3.1. Extraction of Essential Oil

The extraction of essential oil was carried out by boiling 300 g of fresh leaves of *C. winterianus* in water using a 3-litre capacity Clevenger apparatus for 3^½^ h in three replicates. The essential oil produced was measured, collected and treated with anhydrous sodium sulphate (Na_2_SO_4_) to remove excess moisture present and was stored in 4 °C for qualitative and quantitative analysis. 

#### 3.3.2. Essential Oil Analysis 

For the qualitative and quantitative evaluation of the major phytochemical biomarkers, a GC-FID and GC-MS analysis of the essential oil was carried out using the following instrument and GC conditions (Table 6) [69]:

The identification of the major components was done by comparing the retention time of the standard samples (Sigma Aldrich, Germany and Hi-media, India) with the same GC conditions, and the percentages of the compounds were determined by an area normalization method. In GC-MS, the mass spectra of the obtained peaks were identified by comparison with the NIST/WILEY Mass Spectral Library and retention indices within the literature.

#### 3.3.3. Statistical Analysis

MINITAB 16.0 software (Minitab Inc, State College, PA, USA) was used to evaluate the analysis of the variances (ANOVA) for the quantitative data of citronellal, citronellol and geraniol, followed by dendrogram clustering based on complete linkage and Euclidean distance.

## 4. Conclusions

*C. winterianus* is a plant of high industrial importance because of its yielding capacity for essential oil and its compounds of high pharmaceutical importance. Therefore, preservation and conservation is highly recommended for proper utilisation of the crop in breeding programmes. Several breeding techniques, such as molecular, mutation and selection breeding, are the most suitable measures for the development of high-yielding varieties. Successful breeding depends upon genetic diversity and information about the accessions collected from different places. Therefore, diversity based on phytochemical and molecular markers was studied to differentiate the accessions into different clusters. Depending on the requirement of the particular constituents, genotypes could be selected for the development of recombinant lines with desirable characteristics. The recombinant lines may contain more essential oil with a highly valuable lead molecule. The knowledge obtained by this study would also enhance the information regarding the phylogeny of different accessions and would enrich the gene bank. The findings would help in adopting proper conservation strategies by minimizing the risk of extinction of the elite lines. Most importantly, the scientific community, academicians, and the commercial sector would benefit highly by utilizing this research work for plant improvement programmes. 

## Figures and Tables

**Table 1:** Indigenous collection number (IC) and GPS locations of different collected accessions of *C. winterianus* along with their major phytochemical constituents in percentage.

S. No	IC. No.	Collection Site	Citronellal %	Citronellol %	Geraniol %
1	IC-0627004	Tinsukia/Tinsukia/Assam	3.745 ± 0.098	20.433 ± 0.021	41.146 ± 0.058
2	IC-0627019	Senapati/Senapati/Manipur	43.404 ± 0.067	14.935 ± 0.055	20.188 ± 0.041
3	IC-0627018	Senapati/Senapati/Manipur	42.934 ± 0.015	11.264 ± 0.071	22.437 ± 0.034
4	IC-0627017	Churachandpur/Churachandpur/Manipur	42.641 ± 0.053	11.374 ± 0.017	23.270 ± 0.062
5	IC-0627016	Chandel/Chandel/Manipur	41.843 ± 0.067	9.862 ± 0.048	22.323 ± 0.056
6	IC-0627006	Tinsukia/Tinsukia/Assam	43.333 ± 0.019	15.018 ± 0.003	13.339 ± 0.005
7	IC-0627005	Dibrugarh/Dibrugarh/Assam	42.196 ± 0.055	11.234 ± 0.022	18.851 ± 0.037
8	IC-0627003	Titabar/Jorhat/Assam	4.485 ± 0.083	23.000 ± 0.041	39.842 ± 0.075
9	IC-0627002	Titabar/Jorhat/Assam	5.723 ± 0.080	26.389 ± 0.052	31.448 ± 0.005
10	IC-0626993	Tippi/West Kameng/AP	51.450 ± 0.048	10.064 ± 0.087	19.320 ± 0.075
11	IC-0626991	Nongpoh/Ri Bhoi/Meghalaya	42.501 ± 0.063	14.311 ± 0.014	17.301 ± 0.042
12	IC-0627064	Birjhora T.E/Bongaigaon/Assam	19.975 ± 0.051	11.143 ± 0.018	29.912 ± 0.037
13	IC-0627065	Moijonga/Goalpara/Assam	46.511 ± 0.063	13.747 ± 0.027	19.304 ± 0.079
14	IC-0627063	Birjhora T.E/Bongaigaon/Assam	46.022 ± 0.018	11.919 ± 0.046	18.807 ± 0.031
15	IC-0627062	Dejoo T.E/North Lakhimpur/Assam	48.477 ± 0.057	9.245 ± 0.061	20.480 ± 0.028
16	IC-0627058	Siajuli T.E/North Lakhimpur/Assam	47.383 ± 0.063	11.282 ± 0.074	14.979 ± 0.052
17	IC-0627057	Dejoo T.E/North Lakhimpur/Assam	46.658 ± 0.038	10.329 ± 0.140	20.040 ± 0.097
18	IC-0627056	Siajuli T.E/North Lakhimpur/Assam	46.606 ± 0.071	11.425 ± 0.044	17.554 ± 0.086
19	IC-0627052	Tingkhong T.E/Dibrugarh/Assam	46.287 ± 0.083	10.728 ± 0.052	18.898 ± 0.029
20	IC-0627051	Sarojini T.E/Dibrugarh/Assam	46.411 ± 0.046	11.971 ± 0.061	16.671 ± 0.018
21	IC-0627050	Birjhora/Bongaigaon/Assam	42.738 ± 0.022	12.880 ± 0.071	20.242 ± 0.034
22	IC-0627049	Manjha/Karbianglong/Assam	48.744 ± 0.011	12.182 ± 0.058	15.282 ± 0.029
23	IC-0627048	Manjha/Karbianglong/Assam	45.785 ± 0.042	11.419 ± 0.030	20.443 ± 0.057
24	IC-0627047	Manjha/Karbianglong/Assam	40.192 ± 0.057	12.722 ± 0.092	24.037 ± 0.023
25	IC-0627046	Manjha/Karbianglong/Assam	41.200 ± 0.014	8.999 ± 0.052	21.093 ± 0.075
26	IC-0627045	Manjha/Karbianglong/Assam	38.411 ± 0.052	11.272 ± 0.037	24.342 ± 0.049
27	IC-0627044	Manjha/Karbianglong/Assam	37.405 ± 0.043	11.925 ± 0.062	22.823 ± 0.057
28	IC-0627042	Manjha/Karbianglong/Assam	38.166 ± 0.051	11.753 ± 0.009	23.059 ± 0.036
29	IC-0627041	Manjha/Karbianglong/Assam	40.901 ± 0.034	11.049 ± 0.057	18.885 ± 0.044
30	IC-0627040	Manjha/Karbianglong/Assam	40.860 ± 0.052	11.993 ± 0.043	21.321 ± 0.071
31	IC-0627039	Manjha/Karbianglong/Assam	42.306 ± 0.049	12.591 ± 0.027	20.396 ± 0.052
32	IC-0627038	Powai/Tinsukia/Assam	38.345 ± 0.061	11.878 ± 0.033	22.924 ± 0.038
33	IC-0627037	Raidang/Tinsukia/Assam	43.995 ± 0.057	13.729 ± 0.061	22.272 ± 0.041
34	IC-0627036	Sokriting T.E./Tinsukia/Assam	18.938 ± 0.043	12.122 ± 0.025	11.734 ± 0.076
35	IC-0627033	Moranhat/Dibrugarh/Assam	31.997 ± 0.062	12.288 ± 0.059	22.196 ± 0.071
36	IC-0627035	Panitola/Tinsukia/Assam	47.569 ± 0.055	11.116 ± 0.049	18.029 ± 0.038
37	IC-0627034	Bogapani/Tinsukia/Assam	35.657 ± 0.037	10.261 ± 0.082	24.533 ± 0.051
38	IC-0627032	Sepon T.E/Dibrugarh/Assam	39.754 ± 0.021	11.103 ± 0.076	22.976 ± 0.039
39	IC-0627031	Gangabari T.E./Tinsukia/Assam	19.057 ± 0.069	9.474 ± 0.048	38.826 ± 0.082
40	IC-0627028	Angkailongdi/Senapati/Manipur	40.541 ± 0.075	11.978 ± 0.024	22.376 ± 0.061
41	IC-0627030	Tuithapi/Churachandpur/Manipur	46.781 ± 0.043	13.714 ± 0.037	20.406 ± 0.050
42	IC-0627026	Japhou/Chandel/Manipur	44.400 ± 0.019	12.677 ± 0.095	19.164 ± 0.039
43	IC-0627027	Kalika/Imphal East/Manipur	41.479 ± 0.038	11.134 ± 0.042	21.566 ± 0.017
44	IC-0627024	Gumte/East Kameng/AP	49.090 ± 0.013	13.578 ± 0.074	17.144 ± 0.042
45	IC-0627025	Silluk/East Siang/AP	38.012 ± 0.032	10.726 ± 0.056	22.645 ± 0.013
46	IC-0627023	Jangda/Tawang/AP	45.200 ± 0.068	11.486 ± 0.057	19.139 ± 0.065
47	IC-0627022	Boleng/East Siang/AP	36.825 ± 0.024	9.661 ± 0.049	20.483 ± 0.039
48	IC-0627021	Picha/Kurungkumey/AP	42.863 ± 0.055	15.486 ± 008	21.574 ± 0.027
49	IC-0627054	Sarojini T.E/Dibrugarh/Assam	43.105 ± 0.037	14.106 ± 0.029	18.136 ± 0.034
50	IC-0627043	Manjha/Karbianglong/Assam	41.864 ± 0.022	13.929 ± 0.045	23.171 ± 0.017
51	IC-0626995	Itanagar/Itanagar/AP	20.119 ± 0.039	14.112 ± 0.017	35.834 ± 0.045
52	IC-0627060	Ananda T.E/North Lakhimpur/Assam	49.543 ± 0.046	10.153 ± 0.013	18.670 ± 0.082
53	IC-0627055	Birjhora T.E/Bongaigaon/Assam	44.487 ± 0.061	11.503 ± 0.072	12.079 ± 0.042
54	IC-0626994	Jang/Tawang/AP	42.225 ± 0.046	13.442 ± 0.095	17.160 ± 0.052
55	IC-0627059	Dolohat T.E/North Lakhimpur/ Assam	45.059 ± 0.074	13.200 ± 0.024	16.611 ± 0.059
56	IC-0627012	Karbianglong/Karbianglong/Assam	22.730 ± 0.043	11.733 ± 0.027	22.741 ± 0.038
57	IC-0626998	Golaghat/Golaghat/Assam	43.068 ± 0.052	9.637 ± 0.078	21.123 ± 0.014
58	IC-0627013	Umiam/ Ri Bhoi/Meghalaya	21.914 ± 0.024	9.986 ± 0.132	34.194 ± 0.154
59	IC-0627001	Mariani/Jorhat/Assam	42.346 ± 0.092	15.290 ± 0.014	18.762 ± 0.043
60	IC-0627029	Saikot/Churachandpur/Manipur	44.850 ± 0.089	13.011 ± 0.046	22.343 ± 0.057
61	IC-0627009	Nagaon/Nagaon/Assam	2.356 ± 0.048	0.000	0.000
62	IC-0627014	Barpeta/Barpeta/Assam	45.857 ± 0.074	13.245 ± 0.016	19.208 ± 0.061
63	IC-0626996	Rupa/West Kameng/AP	45.015 ± 0.062	12.879 ± 0.047	19.924 ± 0.035
64	IC-0626999	Golaghat/Golaghat/Assam	3.559 ± 0.071	10.532 ± 0.063	22.834 ± 0.054
65	IC-0626997	Golaghat/Golaghat/Assam	2.703 ± 0.083	2.682 ± 0.075	2.967 ± 0.073
66	IC-0627000	Mariani/Jorhat/Assam	40.551 ± 0.069	10.816 ± 0.021	21.960 ± 0.056
67	IC-0627011	Karbianglong/Karbianglong/Assam	21.393 ± 0.007	9.147 ± 0.084	38.883 ± 0.037
68	IC-0627061	Harmoti T.E/North Lakhimpur/Assam	43.561 ± 0.045	9.567 ± 0.063	21.127 ± 0.050
69	IC-0626992	Tippi/West Kameng/AP	40.386 ± 0.037	11.145 ± 0.029	19.420 ± 0.010
70	IC-0627053	Tingkhong T.E/Dibrugarh/Assam	40.309 ± 0.023	10.628 ± 0.031	18.320 ± 0.081
71	IC-0627007	Moran/Dibrugarh/Assam	38.334 ± 0.045	11.773 ± 0.018	20.003 ± 0.063
72	IC-0627008	Dibrugarh/Dibrugarh/Assam	2.578 ± 0.012	0.000	0.000

GPS: global positioning system, IC.: Indigenous collection.

**Table 2:** SSR marker used in the study along with polymorphic and monomorphic alleles, polymorphism percentage, polymorphism information content, marker index and resolving power.

S. No.	Primer Code	Primer Sequence (5ʹ–3ʹ and 3ʹ–5ʹ)	Total Alleles	Polymorphic Alleles	% Polymorphism	PIC	MI	Rp
1	1CM0102	TGCACGGGGAAGAGGAGAGA AACCGAAGCGCAAGAACCCA	4	3	75	0.47	0.35	2.14
2	2CM0304	ACCCCAGCGCGATCCGAGGT AGCGGCGTCTCCAGCCCGAA	3	2	67	0.55	0.37	0.70
3	3CM0506	CGCCGAAGTGGTAGAGGCAA TGCAGGAGGCAGGAGGAGAA	5	5	100	0.69	0.69	3.12
4	4CM0910	CGCCGCCGTACTGCTCCATC GCGGAGGAGACCTGCGGGT	5	4	80	0.58	0.47	2.16
5	5CM1112	GAATCCGATCCATCCATTGG CACGAACACGCGACGCACGA	3	1	33	0.33	0.11	0.01
6	6CM1314	ACTTTGCGTGCGAGGCGAGG CGAGGTCGAGTAGAAGGCGT	3	2	67	0.42	0.28	0.63
7	7CM1516	ACTTTGCGTGCGAGGCGAGG CGAGGTCGAGTAGAAGGCGT	2	1	50	0.50	0.25	0.02
8	8CM1718	CTCGCCGTCGAATCCGCCAT CACTCTCCTCTCCTGGCCCG	2	1	50	0.49	0.25	0.03
9	10CM2122	CTCCCCCTCCTCCTCCTCCC CGACCGGCCGGAATGGATGC	3	2	67	0.66	0.44	0.05
10	12CM2728	ATCTCTTCGCAGATCCACCT GCTGAGACGCGCGGGTCGGA	3	2	67	0.58	0.39	0.51
11	13CM2930	TTTATCCGCGTCCCTAGCTT GCCGCCGGGGTCACAGGTCA	5	3	60	0.57	0.34	0.31
12	14CM3132	GAGAGGATTCCGATACCCTT TCGGCCTCTCGCCCCCCGA	3	3	100	0.50	0.50	2.84
13	15CM3334	CGTGCTCGTGGATCCCCATC CACCGTCGAATCGAATCCAA	5	4	80	0.65	0.52	1.47
14	17CM3738	ATATATCCAGCCAGCCGCAT GGGCCGTGCCGTGCCTCACC	3	2	67	0.16	0.11	0.95
15	18CM3940	CTCCTCGATCTTCCTCTACT TCGACCCCATCACAAATCCA	4	3	75	0.65	0.48	0.84
16	20CM4344	GGGATGATCATCTCCGATGC CCCTTCTCCCACTCTTCTCC	4	3	75	0.67	0.50	0.63
17	21CM038	GGGAATGGGAAGGCAGTTG AGCGCAGCACCACGAGAA	5	3	60	0.39	0.23	1.31
18	22CM075	TGGCTAAGGAAGAATGCTC CAGCGACGTCAATGCCACC	3	2	67	0.54	0.36	0.74
19	25CM003	TGATGCAGTCGCCAAAAAGGATGA ACGCGGCCTTTTTACGGTTCCTG	2	1	50	0.29	0.14	0.85
20	30CM056	GGACACCATTGCTTTTTAGTAGAG CGGAGAAGGACGACAGAGACG	3	3	100	0.52	0.52	1.26
21	31CM154	TGAATAATCGCGGAATGAAGTGGA GCTCAGCACCCTTCACATCTTTAG	4	3	75	0.41	0.31	2.36
22	33CM163	ACGCGGAGCCGACAGAGGATCT GGCAGGGATGGGGGCGTCTGG	2	1	50	0.49	0.25	0.04
23	34CM014	GTTGCATATGCCGGCGACTGTG GCCGGGCGCCATCATTAGTTC	3	3	100	0.34	0.34	1.62
24	35CM007	CCGGGGACATTTGGGACTCG GGCGCCGTCCACAAAGAATC	4	3	75	0.40	0.30	1.32
25	36CM126	ACGAAGCACAGCGGGAAGGTTC CCTGCCGTTGTTCTCCATCCAC	3	2	67	0.41	0.27	0.67
26	38CM168	AACATGTTCAGAAAAGCGCAG CGCCGCTTGCGCTTATGCTCG	2	1	50	0.49	0.25	0.03
27	39CM125	CGATGCAGCGCGAGAGTAGAGA AACCTTCCCGCTGCTTCGTGCA	2	1	50	0.49	0.24	0.05
28	42CM128	CGTTTGCGCGAAGAGCCCTACCC CCGCACGCCATCCCCAACCA	2	1	50	0.47	0.24	0.11
Total			92	65	1907	13.71	9.5	26.77
Average			3.29	2.32	68.10	0.49	0.34	0.96
Standard deviation			1.05	1.09	17.49	0.12	0.14	0.90

PIC: polymorphism information content, MI: marker index, Rp: resolving power.

**Table 3:** Genetic diversity parameters of *C. winterianus* populations based on SSR primers.

S. No.	*C. winterianus*	Mean				Polymorphic %	Ht	Hs	Gst	Nm
		na	ne	h	I					
1.	Pop 1 (Assam)	1.71 ± 0.46	1.42 ± 0.39	0.24 ± 0.20	0.36 ± 0.28	70.65	0.22 ± 0.03	0.16 ± 0.02	0.25	1.48
2.	Pop 2 (Manipur)	1.40 ± 0.49	1.23 ± 0.34	0.13 ± 0.19	0.20 ± 0.27	40.22				
3.	Pop 3 (Meghalaya)	1.18 ± 0.39	1.13 ± 0.28	0.07 ± 0.16	0.11 ± 0.24	18.48				
4.	Pop 4 (Arunachal Pradesh)	1.58 ± 0.50	1.34 ± 0.36	0.20 ± 0.20	0.31 ± 0.28	57.61				
Mean		1.71	1.41	0.24	0.36	70.65				
Standard deviation		0.46	0.38	0.20	0.27					

na: observed number of alleles, ne: effective number of alleles [Kimura and Crow (1964)], h: Nei’s (1973) gene diversity, I: Shannon’s information index [Lewontin (1972)], Ht: total species diversity, Hs: diversity within population, Gst: gene differentiation coefficient, Nm: estimate of gene flow from Gst.

**Table 4:** Eigen value, % variance and cumulative variance of principal component analysis based on SSR marker.

PC	Eigen Value	% Variance	Cumulative Variance
1	1.91	16.09	16.09
2	0.99	8.36	24.45
3	0.69	5.77	30.22
4	0.67	5.61	35.83
5	0.54	4.56	40.31
6	0.53	4.42	44.81
7	0.48	4.07	48.88
8	0.46	3.86	52.74
9	0.40	3.38	56.12
10	0.35	2.96	59.08
11	0.34	2.88	61.96
12	0.30	2.54	64.50
13	0.30	2.49	66.99
14	0.28	2.34	69.33

**Table 5:** AMOVA (analysis of molecular variance) of *C. winterianus* germplasm based on SSR primers.

Source of Variation	Degrees of Freedom (df)	Sum of Squares (SS)	Mean Squares (MS)	Variance Component	Percentage of Total Variance	*p* Value
Among Pops	3	10.223	3.408	0.243	25%	0.255
Within Pops	68	48.374	0.711	0.711	75%	0.045
Total	71	58.597		0.954	100%	

Level of significance based on 999 permutations; Pop: population; *p* value: level of marginal significance.

**Table 6:** The optimum conditions for GC-MS and GC-FID analysis in *C. winterianus* essential oil.

	GC-MS	GC-FID
Instrument	TRACE Ultra Gas Chromatograph coupled with an ISQ Mass Spectrometer	Thermo Scientific TRACE 1110
Column	TG WAX/MS (60 m × 0.25mm i.d)	TG WAX/MSA (60 m × 0.25mm i.d)
Film thickness	0.25 μm	0.25 μm
Running conditions	40 °C for 2 min 250 °C at 5 °C/min 300 °C at 30 °C/min for 10 min	Initial temperature: 50 °C Oven temperature: 300 °C Injector temperature: 280 °C FID temperature: 310 °C
Sample injection	1 μL	1 μL
Carrier gas	Helium (1mL min^−1^)	Nitrogen (0.30 mL min^−1^)

**Figure A1:**
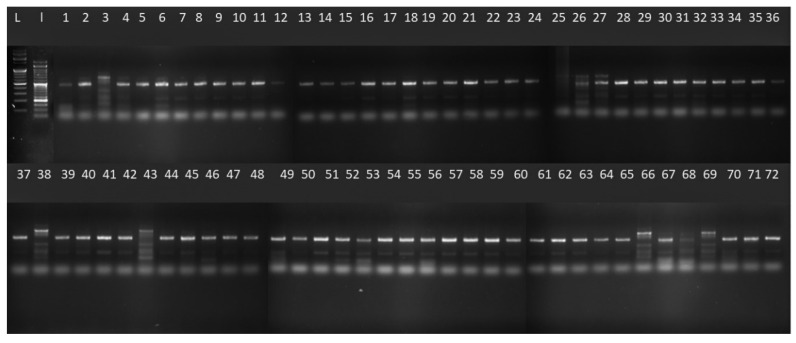
Gel image of primer 15CM3334 depicting diversity in *C. winterianus* germplasm.

## Data Availability

Data is contained within the article.

## Appendix A

**Table A1:** The allele frequency divergences among and within (bold) populations were computed using point estimates of P.

	Pop I	Pop II	Pop III	Pop IV	Pop V	Pop VI
Pop I	**0.1215**	0.1589	0.1377	0.0620	0.1144	0.0999
Pop II		**0.1774**	0.0980	0.1793	0.0977	0.0804
Pop III			**0.2395**	0.1171	0.0842	0.0913
Pop IV				**0.1386**	0.1240	0.1124
Pop V					**0.2296**	0.0707
Pop VI						**0.1666**

## References

[1] Bhattacharya S., Bandopadhyay T.K., Ghosh P.D. (2010). Efficiency of RAPD and ISSR markers in assessment of molecular diversity in elite germplasms of *Cymbopogon winterianus* across West Bengal, India. Emir. J. Food Agric..

[2] Paterson A.H., Tanksley S.D., Sorrells M.E. (1991). DNA markers in plant improvement. Adv. Agron..

[3] Perrino E.V., Perrino P. (2020). Crop wild relatives: Know how past and present to improve future research, conservation and utilization strategies, especially in Italy: A review. Resour. Crop Evol..

[4] Adhikari S., Saha S., Bandyopadhyay T.K., Ghosh P. (2015). Efficiency of ISSR marker for characterization of *Cymbopogon* germplasms and their suitability in molecular barcoding. Plant Syst. Evol..

[5] Lal M., Munda S., Dutta S., Pandey S.K. (2020). Identification of a novel germplasm (Jor Lab L-9) of lemon grass (*Cymbopogon khasianus*) rich in methyl eugenol. Crop Breed. Appl. Biotechnol..

[6] Dutta S., Munda S., Lal M., Bhattacharyya P.R. (2016). A short review on Chemical composition, therapeutic use and enzyme inhibition activities of *Cymbopogon* species. Indian J. Sci. Technol..

[7] Lal M., Dutta S., Munda S., Pandey S.K. (2018). Novel high value elemicin-rich germplasm of Lemon grass (*Cymbopogn khasianus* (hack) Staf (ex Bor) from North East India. Ind. Crops Prod..

[8] Deka H., Deka S., Baruah C.K., Das J., Hoque S., Sarma N.S. (2011). Vermicomposting of distillation waste of citronella plant (*Cymbopogon winterianus* Jowitt.) employing *Eudrilus eugeniae*. Bioresour. Technol..

[9] Dey T., Saha S., Ghosh P.D. (2015). Somaclonal variation among somatic embryo derived plants-Evaluation of agronomically important somaclones and detection of genetic changes by RAPD in *Cymbopogon winterianus*. S. Afr. J. Bot..

[10] Lal M., Dutta S., Bhattacharyya P.R. (2016). Development of a new superior variety (Jor Lab C-5) of Java Citronella with characteristics of stable and high oil yield. Ann. Biol..

[11] Lal M. (2018). Jor Lab C-5 (IC0619027; INGR16021), a Java citronella (*Cymbopogon winterianus* Jowitt) germplasm with high herbage yield with high essential oil content. Indian J. Plant Genet. Resour..

[12] Simic A., Rancic A., Sokovic M.D., Ristic M., Grujic-Jovanovic S., Vukojevic J., Marin P.D. (2008). Essential oil composition of *Cymbopogon winterianus* and *Carum carvi* and their antimicrobial activities. Pharm. Biol..

[13] Lawless J. (2002). The Encyclopaedia of Essential Oils.

[14] Quintans-Junior L.J., Souza T.T., Leite B.S., Lessa N.M., Bonjardim L.R., Santos M.R., Alves P.B., Blank A.F., Antoniolli A.R. (2008). Phytochemical screening and anticonvulsant activity of *Cymbopogon winterianus* Jowitt (Poaceae) leaf essential oil in rodents. Phytomedicine.

[15] Munda S., Dutta S., Pandey S.K., Sarma N., Lal M. (2019). Antimicrobial activity of essential oils of medicinal and aromatic plants of the North east India: A biodiversity hot spot. J. Essent. Oil Bear. Pl..

[16] Ganjewala D. (2009). *Cymbopogon* essential oils: Chemical compositions and bioactivities. Int. J. Essent. Oil Ther..

[17] Ganjewala D., Gupta A.K. (2016). Lemongrass (*Cymbopogon flexuosus* Steud.) wats essential oil: Overview and biological activities. Essent. Oils II RPMP.

[18] Kumar V., Roy B.K. (2018). Population authentication of the traditional medicinal plant *Cassia tora* L. based on ISSR markers and FTIR analysis. Sci. Rep..

[19] Perrino E.V., Valerio F., Jallali S., Trani A., Mezzapesa G.N. (2021). Ecological and biological properties of *Satureja cuneifolia* and *Thymus spinulosus* Ten.: Two wild officinal species of conservation concern in Apulia (Italy). A preliminary survey. Plants.

[20] Cassel E., Vargas R.M.F. (2006). Experiments and modeling of the *Cymbopogon winterianus* essential oil extraction by steam distillation. J. Mex. Chem. Soc..

[21] Sousa D.P., Gonclaves J.C.R., Quintans J.L., Cruz J.S., Araujo D.A.M., Almeida R.N. (2006). Study of anticonvulsant effect of citronellol, a monoterpene alcohol, in rodents. Neurosci. Lett..

[22] Gogoi R., Loying R., Sarma N., Munda S., Pandey S., Lal M. (2018). A comparative study on antioxidant, anti-inflammatory, genotoxicity, anti-microbial activities and chemical composition of fruit and leaf essential oils of *Litsea cubeba* Pers. from North-east India. Ind. Crops Prod..

[23] Sultanbawa Y., Preedy V.R. (2016). Essential oils in food application: Australian aspects. Essential Oils in Food Preservation, Flavor and Safety.

[24] Silva Y.M.S., Silva M.T.A., Sampaio P.A., Quintans J.S.S., Qunitans-Junior L.J., Ribeiro L.A.A. (2014). Relaxant effect of carvacrol, citronellal and p-cymene, monoterpenes present in *Thymus* and *Cymbopogon* species, in guinea-pig trachea: A comparative study. J. Med. Plant Res..

[25] Burdock G.A. (2010). Fenaroli’s Handbook of Flavor Ingredients.

[26] Chen W., Viljoen A.M. (2010). Geraniol-A review of a commercially important fragrance material. S. Afr. J. Bot..

[27] Lal M., Borah A., Pandey S.K. (2019). Identification of a new high geraniol rich variety “Jor Lab L-15” of Lemongrass [*Cymbopogon khasianus* (Hack) Stapf (ex Bor)]. J. Essent. Oil Bear. Pl..

[28] Surburg H., Panten J. (2006). Common Fragrance and Flavor Materials: Preparation, Properties and Uses.

[29] Heiba H.I., Rizk A.M. (1986). Constituents of *Cymbopogon* species. Qatar Univ. Sci. Bull..

[30] Munda S., Lal M., Singh B. (2020). Cymbopogon winterianus Jowitt ex Bor, a Hub for various industrial and pharmaceutical applications. Botanical Leads for Drug Discovery.

[31] Shasany A.K., Lal R.K., Patra N.K., Darokar M.P., Garg A., Kumar S., Khanuja S.P.S. (2000). Phenotypic and RAPD diversity among *Cymbopogon winterianus* Jowitt accessions in relation to *Cymbopogon nardus* Rendle. Genet. Resour. Crop Evol..

[32] Kumar J., Verma V., Goyal A., Shahi A.K., Sparoo R., Sangwan R.S., Qazi1 G.N. (2009). Genetic diversity analysis in *Cymbopogon* species using DNA markers. Plant Omics.

[33] Munda S., Dutta S., Lal M. (2020). Variability estimation and genetic divergence in *Cymbopogon winterianus* for development of superior genotype. Agron. J..

[34] Gupta A.K., Ganjewala D. (2015). A study on developmental changes in essential oil content and composition in *Cymbopogon flexuosus* cultivar Suvarna. Acta Biol. Szeged..

[35] Baruah J., Gogoi B., Das K., Ahmed N.M., Sarmah D.K., Lal M., Bhau B.S. (2016). Genetic diversity study amongst *Cymbopogon* species from NE-India using RAPD and ISSR markers. Ind. Crops Prod..

[36] Sangwan N.S., Yadav U., Sangwan R.S. (2001). Molecular analysis of genetic diversity in elite Indian cultivars of essential oil trade types of aromatic grasses (*Cymbopogon* species). Plant Cell Rep..

[37] Herrera M.R., Ghislain M. (2013). Robust and inexpensive SSR markers analyses using LI-COR DNA analyzer. Microsatellites.

[38] Naghavi M.R., Mardi M., Pirseyedi S.M., Kazemi M., Potki P., Ghaffari M.R. (2007). Comparison of genetic variation among accessions of *Aegilops tauschii* using AFLP and SSR markers. Genet. Resour. Crop. Evol..

[39] Henkrar F., El-Haddoury J., Ouabbou H., Nsarellah N., Iraqi D., Bendaou N., Udupa S.M. (2016). Genetic diversity reduction in improved durum wheat cultivars of Morocco as revealed by microsatellite markers. Sci. Agric..

[40] Abbasov M., Akparov Z., Gross T., Babayeva S., Izzatullayeva V., Hajiye E., Rustamov K., Gross P., Tekin M., Akar T. (2018). Genetic relationship of diploid wheat (*Triticum* spp.) species assessed by SSR markers. Genet. Resour. Crop Evol..

[41] Song Q.J., Shi J.R., Singh S., Fickus E.W., Costa J.M., Lewis J., Gill B.S., Ward R., Cregan P.B. (2005). Development and mapping of microsatellite (STMS) markers in wheat. Theor. Appl. Genet..

[42] Baruah J., Pandey S.K., Sarmah N., Lal M. (2019). Assessing molecular diversity among high capsaicin content lines of *Capsicum chinense* Jacq. using simple sequence repeat marker. Ind. Crops Prod..

[43] Hajiyev E.S., Akparov Z.I., Aliyev R.T., Saidova S.V., Izzatullayeva V.I., Babayeva S.M., Abbasov M.A. (2015). Genetic polymorphism of durum wheat (*Triticum durum* Desf.) accessions of Azerbaijan. Russ. J. Genet..

[44] Abbasov M., Brueggeman R., Raupp J., Akparov Z., Aminov N., Bedoshvili D., Gross T., Gross P., Babayeva S., Izzatullayeva V. (2019). Genetic diversity of *Aegilops* L. species from Azerbaijan and Georgia using SSR markers. Genet. Resour. Crop Evol..

[45] Kaur P., Pandeya D.K., Gupta R.C., Dey A. (2019). Assessment of genetic diversity among different population of five *Swertia* species by using molecular and phytochemical markers. Ind. Crops Prod..

[46] Hyun D.Y., Gi G.Y., Sebastin R., Cho G.T., Kim S.H., Yoo E., Lee S., Son D.M., Lee K.J. (2020). Utilization of phytochemical and molecular diversity to develop a target-oriented core collection in tea germplasm. Agronomy.

[47] Qaderi A., Omidi M., Pour-Aboughadareh A., Poczai P., Shaghaghi J., Mehrafarin A., Nohooji M.G., Etminan A. (2019). Molecular diversity and phytochemical variability in the Iranian poppy (*Papaver bracteatum* Lindl.): A baseline for conservation and utilization in future breeding programmes. Ind. Crops Prod..

[48] Ray A., Jena S., Haldar T., Sahoo A., Kar B., Patnaik J., Ghosh B., Panda P.C., Mahapatra N., Nayak S. (2019). Population genetic structure and diversity analysis in *Hedychium coronarium* populations using morphological, phytochemical and molecular markers. Ind. Crops Prod..

[49] Saikia D., Dutta S., Ghosh S., Lal M., Bhau B.S. (2015). RAPD and ISSR based intra-specific molecular genetic diversity analysis of *Cymbopogon flexuosus* L. Stapf with a distinct correlation of morpho-chemical observations. Res. J. Biotechol..

[50] Lal N., Awasthi S.K. (2015). A comparative assessment of molecular marker assays (AFLP and RAPD) for *Cymbopogon* germplasm characterization. World J. Pharm. Res..

[51] Bishoyi A.K., Sharma A., Kavane A., Geetha K.A. (2016). Varietal discrimination and genetic variability analysis of *Cymbopogon* using RAPD and ISSR markers analysis. Appl. Biochem. Biotechnol..

[52] Paw M., Borah A., Pandey S.K., Baruah J., Begum T., Lal M. (2021). Simple sequence repeat marker based genetic diversity assessment amongst high essential oil yielding lines of *Curcuma caesia* Roxb. Genet. Resour. Crop Evol..

[53] Botstein D., White R.L., Skolnick M., Davis R.W. (1980). Construction of a genetic linkage map in man using restriction fragment length polymorphisms. Am. J. Hum. Genet..

[54] Slatkin M. (1987). Gene flow and geographic structure of natural populations. Science.

[55] Zheng W.H., Zhuo Y., Liang L., Ding W.Y., Liang L.Y., Wang X.F. (2015). Conservation and population genetic diversity of *Curcuma wenyujin* (Zingiberaceae), a multifunctional medicinal herb. Genet. Mol. Res..

[56] Saitou N., Nei M. (1987). The neighbour joining method: A new method for reconstructing phylogenetic trees. Mol. Biol. Evol..

[57] Harsono T., Pasaribu N., Fitmawati F., Sobir S., Prasetya E. (2018). Genetic variability and classification of Gandaria (*Bouea*) in Indonesia based on inter simple sequence repeat (ISSR) markers. SABRAO J. Bred. Genet..

[58] Wold S., Esbensen K., Geladi P. (1987). Principal component analysis. Chemometr. Intell. Lab. Syst..

[59] Luo C., Chen D., Cheng X., Liu H., Li Y., Huang C. (2018). SSR analysis of genetic relationship and classification in *Chrysanthemum* germplasm Collection. Hortic. Pl. J..

[60] Rajwade A.V., Arora R.S., Kadoo N.Y., Harsulkar A.M., Ghorpade P.B., Gupta V.S. (2010). Relatedness of Indian flax genotypes (*Linum usitatissimum* L.): An inter-simple sequence repeat (ISSR) primer assay. Mol. Biotechnol..

[61] Excoffier L., Smouse P.E., Quattro J.M. (1992). Analysis of molecular variance inferred from metric distances among DNA haplotypes: Application to human mitochondrial DNA restriction data. Genetics.

[62] Grunwald N.J., Everhart S.E., Knaus B.J., Kamvar Z.N. (2017). Best practices for population genetic analyses. Phytopathology.

[63] Hogbin P.M., Peakall R. (1999). Evaluation of the conservation of genetic research to the management of endangered plant. Zieriaprostrata Conserv. Biol..

[64] Khanlou K.M., Vandepitte K., Asl L.K., Bockstaele E.V. (2011). Towards an optimal sampling strategy for assessing genetic variation within and among white clover (*Trifolium repens* L.) cultivars using AFLP. Genet. Mol. Biol..

[65] Young A., Merriam H., Warwick S. (1993). The effects of forest fragmentation on genetic variation in *Acer saccharum* Marsh. (sugar maple) populations. Heredity.

[66] Flather C.H., Bevers M. (2002). Patchy reaction-diffusion and population abundance: The relative importance of habitat amount and arrangement. Am. Nat..

[67] Verma R.S., Singha S., Padalia R.C., Tandon S., Venkatesh K.T., Chauhan A. (2019). Essential oil composition of the sub-aerial parts of eight species of *Cymbopogon* (Poaceae). Ind. Crops Prod..

[68] Sangwan R.S., Sangwan N.S., Kumar S., Dwivedi S., Kukreja A.K., Sharma J.R., Bagchi G.D. (2000). Metabolic analysis of oil-chemotypic diversity in *Cymbopogons*. Cymbopogons: The Aromatic Grass Monograph.

[69] Munda S., Sarma N., Lal M. (2020). G × E interaction of 72 accessions with three year evaluation of *Cymbopogon winterianus* Jowitt. using regression coefficient and Additive Main effects and Multiplicative Interaction model (AMMI). Ind. Crops Prod..

[70] Milbourne D., Meyer R., Bradshaw J.E., Baird E., Bonar N., Provan J., Powell W., Robbie W. (1997). Comparison of PCR-based marker systems for the analysis of genetic relationships in cultivated potato. Mol. Breed..

[71] Prevost A., Wilkinson M.J. (1999). A new system of comparing PCR primers applied to ISSR fingerprinting of potato cultivars. Theor. Appl. Genet..

[72] Yeh F.C., Yang R.C., Boyle T. (2000). POPGENE 1.32: A Free Program for the Analysis of Genetic Variation among and within Populations Using Co-Dominant and Dominant Markers. https://agris.fao.org/agris-search/search.do?recordID=GB2012106241.

[73] Peakall R., Smouse P.E. (2006). GenAlEx 6: Genetic analysis in Excel. Population genetic software for teaching and research. Mol. Ecol. Notes.

